# Prognostic assessment capability of a five-gene signature in pancreatic cancer: a machine learning based-study

**DOI:** 10.1186/s12876-023-02700-y

**Published:** 2023-03-11

**Authors:** Xuanfeng Zhang, Lulu Yang, Dong Zhang, Xiaochuan Wang, Xuefeng Bu, Xinhui Zhang, Long Cui

**Affiliations:** 1 Center of Hepatobiliary Pancreatic Disease, XuZhou Central Hospital, Jiangsu, People’s Republic of China; 2 Center of Hepatobiliary Pancreatic Disease, The Affiliated Xuzhou Hospital of Medical School of Southeast University, No.199 Jiefang South Road, Xuzhou, Jiangsu, People’s Republic of China; 3 Department of Radiology, XuZhou Central Hospital, Jiangsu, People’s Republic of China; 4 Department of Radiology, The Affiliated Xuzhou Hospital of Medical School of Southeast University, Jiangsu, People’s Republic of China; 5 Bengbu Medical College, Anhui, People’s Republic of China; 6 Department of General Surgery, Affiliated People’s Hospital of Jiangsu University, Zhenjiang, Jiangsu, People’s Republic of China

**Keywords:** Pancreatic cancer, Bioinformatics, Prognosis, Gaussian finite mixture model, RNA-seq, Machine learning

## Abstract

**Background:**

A prognostic assessment method with good sensitivity and specificity plays an important role in the treatment of pancreatic cancer patients. Finding a way to evaluate the prognosis of pancreatic cancer is of great significance for the treatment of pancreatic cancer.

**Methods:**

In this study, GTEx dataset and TCGA dataset were merged together for differential gene expression analysis. Univariate Cox regression and Lasso regression were used to screen variables in the TCGA dataset. Screening the optimal prognostic assessment model is then performed by gaussian finite mixture model. Receiver operating characteristic (ROC) curves were used as an indicator to assess the predictive ability of the prognostic model, the validation process was performed on the GEO datasets.

**Results:**

Gaussian finite mixture model was then used to build 5-gene signature (ANKRD22, ARNTL2, DSG3, KRT7, PRSS3). Receiver operating characteristic (ROC) curves suggested the 5-gene signature performed well on both the training and validation datasets.

**Conclusions:**

This 5-gene signature performed well on both our chosen training dataset and validation dataset and provided a new way to predict the prognosis of pancreatic cancer patients.

**Supplementary Information:**

The online version contains supplementary material available at 10.1186/s12876-023-02700-y.

## Introduction

Pancreatic cancer (PC) has one of the worst prognoses among malignant tumors, the overall 5-year survival rate of patients with pancreatic cancer is less than 5% [1]. Due to atypical symptoms, no sensitive early diagnostic biomarkers, and exceptional anatomical structures, only about 20% of patients at diagnosis are on the verge of being resectable by surgery [2]. Investigators are currently pursuing a comprehensive medical treatment plan, which includes immunotherapy, targeted drugs, radiotherapy, and chemotherapy [3]. Due to tumor heterogeneity, different patients respond differently to the same treatment regimen. This requires clinicians to adjust treatment regimens based on each patient's response during treatment. Therefore, a prognostic assessment method with good sensitivity and specificity plays an important role in the treatment of patients.

In prognostic assessment, the use of prognosis-related gene expression is better than the use of patient clinical characteristics [4]. The development of next-generation sequencing technology and gene chip technology provides a convenient, accurate, and inexpensive way for the detection of prognosis-related genes. [5]. A growing number of researchers tend to use next-generation sequencing or gene chips to detect prognosis-related genes, then a prognostic model is established to guide the treatment of patients [6]. The mathematical modeling process includes the use of logistic regression, Poisson regression, Cox regression, lasso regression, and ridge regression [7]. The combined use of these bioinformatic modeling approaches can significantly improve the specificity and sensitivity of prognostic models.

Here, we obtained differential genes (DEGs) using the TCGA-PAAD dataset, GTEx and two GEO datasets. For further analysis, we normalized and de-batched all datasets. Eight prognosis-related genes were screened in TCGA-PAAD dataset using univariate Cox regression and lasso regression. The eight genes were permuted and combined, and the AUC value of each combination was calculated separately. The optimal AUC value is then screened using the Gaussian model and validated in the validation set. Ultimately, we found five genes that were excellent in evaluating the prognosis of pancreatic cancer patients in both the training set and validation set.

## Materials and methods

### Data collection and processing

The gene expression data were obtained from four public datasets, including TCGA, Pancreatic Cancer (PAAD) (*n* = 182), GTEx (*n* = 167), GSE62452 (*n* = 130) and GSE28735 (*n* = 90). PAAD was downloaded from UCSC Xena (http://xena.ucsc.edu/), the expression data was normalized to log2(FPKM + 1). GTEx pancreatic cancer expression data was downloaded from UCSC Xena (http://xena.ucsc.edu/) and was normalized to log2(FPKM + 0.001). GSE62452 and GSE28735 were downloaded from GEO database (https://www.ncbi.nlm.nih.gov/geo/), the expression data was normalized by RMA. The expression levels of TCGA-PAAD and GTEx were rescaled using FPKM as the unit of measure for subsequent analyses. Differentially expressed genes (DEGs) was identified using Limma package (version 3.51.8) in R. The cut-off value was set to |logFC|> 1 and *p* value < 0.05.

### Screening for prognosis related DEGs

The univariate Cox regression, LASSO Cox regression models were performed with survival (version 3.1.1) and glmnet (version 4.1.4) R packages. The data screening process was conducted on TCGA-PAAD dataset.

### Data normalization and removing batch effects

The TCGA-PAAD gene expression values were then transformed to FPKM. FPKM were then transformed to TPM. After removing the mRNAs with low expression levels, the TCGA expression level were closer to gene chips. ComBat algorithm which is included in the sva R package (version 3.44.0) was used to remove batch effects.

### Gaussian mixture model

All selected prognosis-related genes were permuted and combined, and used multivariate Cox regression analysis to model, respectively. Calculated AUC for each model separately. AUC value was used as the classification basis and classification was conducted with model-based hierarchical agglomerative clustering which is based on Gaussian finite mixture model. This process used the mclust R package (version 5.4.9) which is a contributed R package for model-based clustering, classification, and density estimation based on finite normal mixture modelling. The associations between prognosis-related gene expression levels and survival information were estimated by the Kaplan–Meier method. The cut-off value was calculated with Kaplan–Meier method.

### Statistical analyses

All the statistical analysis was performed in R software (version 4.2.0). In the survival analysis, Cox proportional hazards regression and Kaplan–Meier analysis were used. We conducted paired t tests on paired samples. All statistical tests with a p-value of less than 0.05 were considered significant.

## Results

### Identification of DEGs in TCGA, GTEx, GSE62452 and GSE28735

**Fig. 1:**
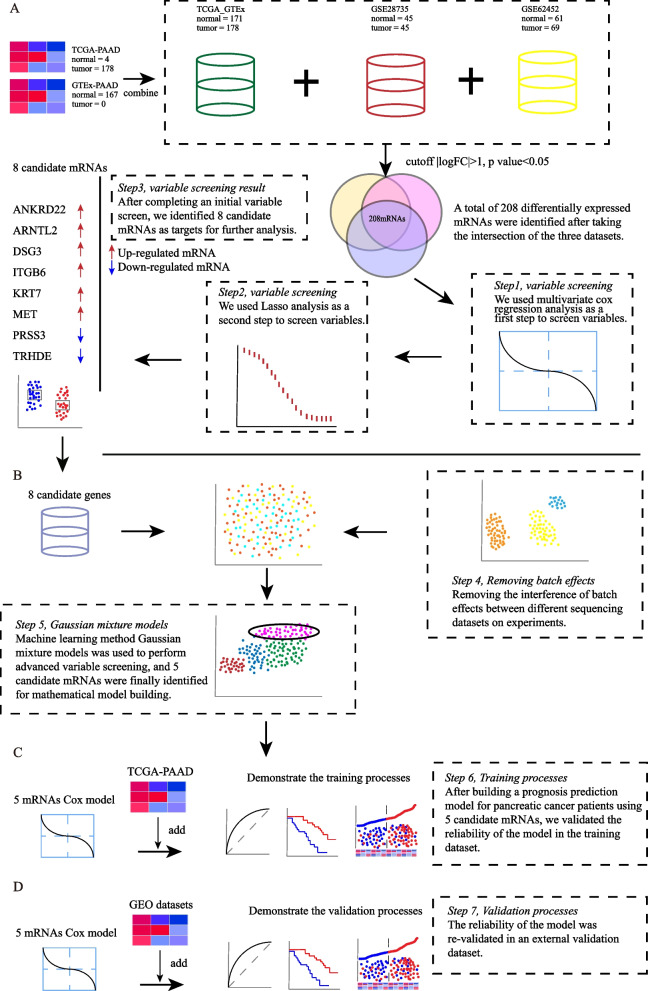
Flowchart for the identification of 5-gene signature. **A** Eight genes associated with the prognosis of pancreatic cancer patients were screened by univariate cox regression and lasso regression. **B** The 5-gene signature was built with using of Gaussian finite mixture model. **C** The processes of training. **D** The processes of validation

This study was conducted according to the flowchart in Fig. 1. TCGA-PAAD, GTEx, GSE62452, and GSE28735 were used to identify DEGs. Because of the lack of normal samples in TCGA-PAAD data, we combined TCGA-PAAD and GTEx-PAAD for analysis. The cut-off value is |logFC|> 1 and *p*-value < 0.05. As shown in Fig. 1A, there are 178 tumor samples and 4 normal samples in TCGA-PAAD dataset. 167 normal samples in GTEx-PAAD dataset, 45 tumor samples and 45 normal samples in GSE28735, 69 tumor samples and 61 normal samples in GSE62452. The TCGA-PAAD and GTEx-PAAD are RNA-seq data, the expression data in them were normalized by FPKM. GSE28735 and GSE62452 share the same platform which ID is GPL6244. The expression data in the array chips was normalized into RAM. Limma R package was used for differential expression analysis. After differential expression analysis, 208 mRNAs were selected by taking the intersection of these datasets (Fig. 1A).

These results suggested that 208 mRNAs have the same expression trends in different datasets (TCGA-PAAD, GTEx-PAAD, GSE62452, and GSE28735). Thus, these initially screened genes might be a promising parameter in patients with pancreatic cancer.

### Identification of prognosis related genes

Firstly, univariate Cox analysis was performed by using TCGA-PAAD expression and clinical data. After excluding genes with P values greater than 0.05, 100 prognostic related genes were screened. To further conduct variable selection and regularization, lasso-penalized Cox analysis was used (Supplementary Fig. 2). The complexity adjustment in the lasso regression algorithm refers to controlling the complexity of the model through a series of parameters to avoid overfitting. As shown in Fig. 1A and Table 1, total 8 candidate genes were selected, which were prognosis related genes. Among these genes, ankyrin repeat domain 22 (ANKRD22), aryl hydrocarbon receptor nuclear translocator like 2 (ARNTL2), desmoglein 3 (DSG3), integrin subunit beta 6 (ITGB6), keratin 7 (KRT7), MET proto-oncogene receptor tyrosine kinase (MET) are up-regulated. The remaining two genes, serine protease 3 (PRSS3) and thyrotropin releasing hormone degrading enzyme (TRHDE), are downregulated (Fig. 1A). The expression trends of these 8 genes were consistent in the 3 datasets.

**Table 1:** Table of differential analysis results for 8 candidate genes in the 3 datasets

TCGA and GTEx				GSE62452				GSE28735			
**Gene**	**log(fold-change)**	p**-value**	**change**	**Gene**	**log(fold-change)**	p**-value**	**change**	**Gene**	**log(fold-change)**	p**-value**	**change**
ANKRD22	1.28	4.38E-22	up	ANKRD22	1.14	2.78E-08	up	ANKRD22	1.58	1.10E-10	up
ARNTL2	1.94	2.39E-54	up	ARNTL2	1.08	3.90E-12	up	ARNTL2	1.26	9.10E-11	up
DSG3	1.14	6.86E-24	up	DSG3	1.04	1.97E-06	up	DSG3	1.01	1.17E-04	up
ITGB6	1.91	1.80E-37	up	ITGB6	2.02	2.66E-13	up	ITGB6	2.08	2.98E-11	up
KRT7	2.42	6.59E-36	up	KRT7	1.49	3.49E-10	up	KRT7	1.68	1.27E-08	up
MET	2.45	2.22E-49	up	MET	1.35	2.14E-13	up	MET	1.48	5.01E-12	up
PRSS3	-4.86	2.34E-47	down	PRSS3	-1.25	4.34E-06	down	PRSS3	-1.21	1.02E-04	down
TRHDE	-1.46	6.39E-48	down	TRHDE	-1.61	1.77E-08	down	TRHDE	-1.68	5.25E-07	down

These results confirmed that 8 candidate genes were associated with the prognosis of pancreatic cancer patients. Further analysis of 8 candidate genes might lead to better tools for assessing prognosis.

### Removing batch effects

**Fig. 2:**
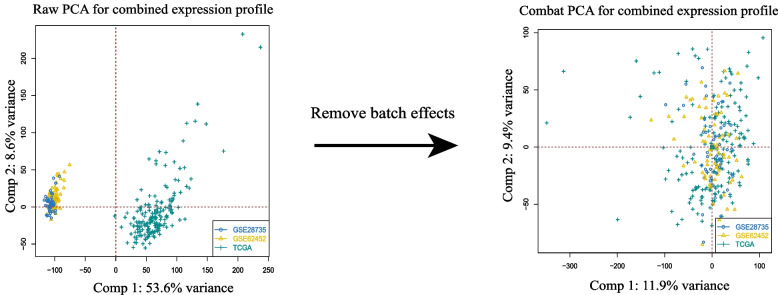
PCA diagrams for the sample distribution of the 3 datasets before and after removing batch effects

**Fig. 3:**
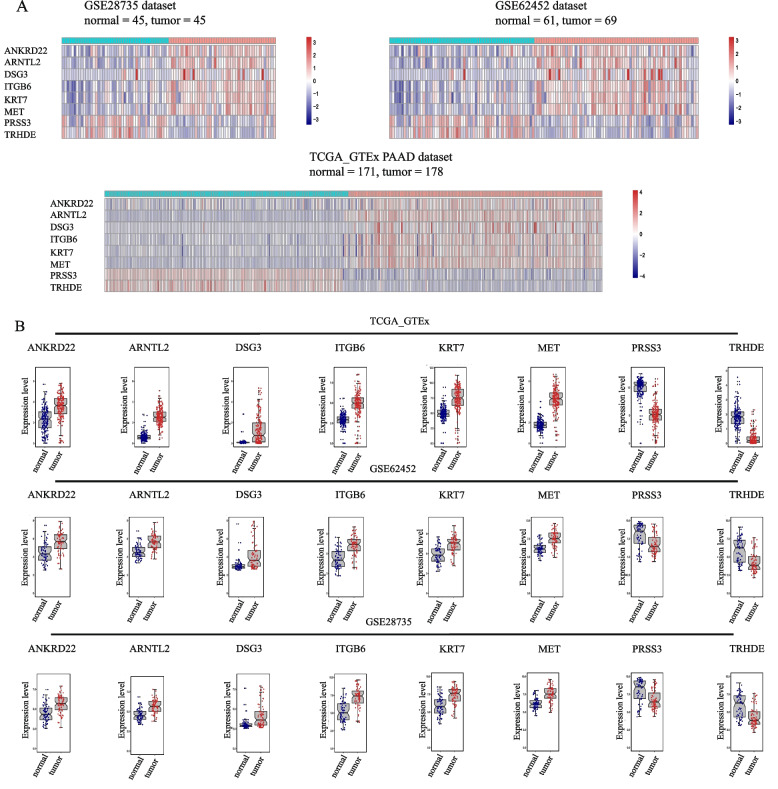
The expression levels of 8 candidate genes. **A** Heatmap. **B** Boxplot

Due to various factors such as experimental personnel, technology, environment, time point, chip processing, etc., the chip expression matrix contains differences in nonbiological factors. Especially when two or more datasets are integrated, even on the same platform, the integration is even more magnified. After adjusting the magnitude of TCGA-PAAD, we used the combat R function to remove the batch effects of these datasets (TCGA, GTEx, GSE62452, GSE28735). As shown in Fig. 2, after de-batching effects, the expression data of the 3 datasets were merged. The expression heatmaps and boxplots generated from the datasets after the batch effect has been removed are shown in Fig. 3.

These results suggested that 8 candidate genes had the same expression level in the 3 datasets.

### Gaussian mixture and Multivariate cox regression model

**Fig. 4:**
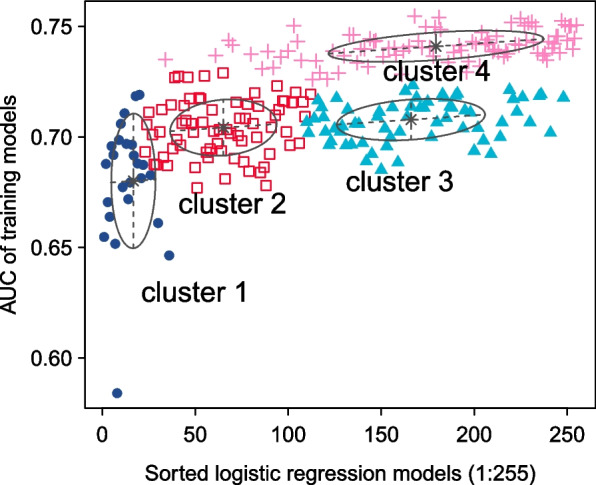
The diagram of gaussian mixture model cluster analysis result. There are 4 clusters of 255 combinations

**Fig. 5:**
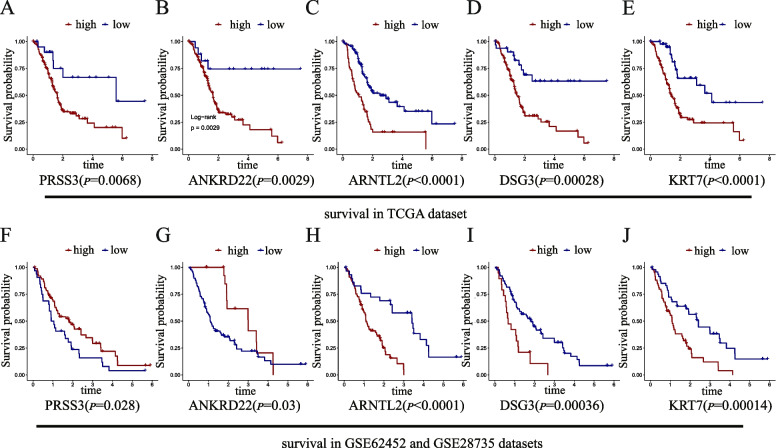
Kaplan–Meier analysis estimates the OS of pancreatic cancer patients according to the expression levels of genes contained in 5-gene signature. **A** PRSS3 in TCGA. **B** ANKRD22 in TCGA. **C** ARNTL2 in TCGA. **D** DSG3 in TCGA. **E** KRT7 in TCGA. **F** PRSS3 in GSE. **G** ANKRD22 in GSE. **H** ARNTL2 in GSE. **I** DSG3 in GSE. **J** KRT7 in GSE

**Fig. 6:**
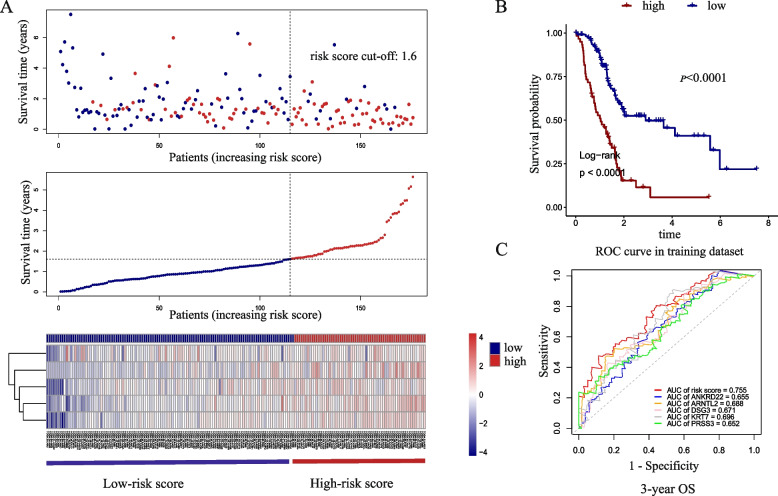
Survival predictive value of the 5-gene signature in TCGA-PAAD patients (training dataset). **A** Patients in TCGA-PAAD dataset were divided into high-risk score and low-risk score groups according to the risk scores calculated with 5-gene signature. **B** Kaplan–Meier analysis estimates the OS of high-risk and low-risk groups in training dataset. **C** ROC curves for 3-year OS by the combined or respective genes

To further fit the model and predict patient prognosis more accurately, we conducted a classification based on Gaussian finite mixture model. After permutation and combination of 8 genes, a total of 255 multivariate Cox regression models were generated. All these models were evaluated for prediction accuracy using AUC. As shown in Fig. 4, 255 models were divided into 4 clusters, and cluster 4 had the highest AUCs. Model 168 that contained in cluster 4 had the highest AUC value of 0.755 (Supplementary table 1). The 5 genes contained in model 168 are ANKRD22, ARNTL2, DSG3, KRT7, and PRSS3. The results of multivariate regression analysis are shown in Table 2. Multivariate regression analysis showed that ANKRD22 (HR: 1.22, 95% CI 0.96–1.55; *P* = 0.098), ARNTL2 (HR: 1.71, 95% CI 1.19–2.46; *P* = 0.003), DSG3 (HR: 1.15, 95% CI 1.01–1.31; *P* = 0.029), KRT7 (HR: 1.19, 95% CI 0.97–1.46; *P* = 0.0093), PRSS3 (HR: 1.23, 95% CI 1.04–1.44; *P* = 0.013) were prognostic factors in pancreatic cancer patients. Survival analysis was performed on this 5-mRNA signature using TCGA training dataset and GSE validation datasets. Kaplan–Meier curves are shown in Fig. 5. According to the multivariate cox regression analysis results, the calculation formula of risk score is [0.19956 * Exp(ANKRD22)] + [0.538767 * Exp(ARNTL2)] + [0.141913 * Exp(DSG3)] + [0.17315 * Exp(KRT7)] + [0.205059 * Exp(PRSS3)]. The best cut-off value was calculated based on Kaplan–Meier analysis. The calculating result of cut-off value is shown in Supplementary Fig. 1A. By dividing the risk score according to its cut-off value (cut-off = 1.6), 176 patients were stratified into high-risk (*n* = 61) and low-risk (*n* = 115) groups (Fig. 6A). Kaplan–Meier curves showed that high-risk patients had worse survival outcome (*P* < 0.0001) (Fig. 6B). Additional ROC curves revealed that 5-mRNA signature model had the best AUC value (0.755), compared to ANKRD22 (0.655), ARNTL2 (0.688), DSG3 (0.671), KRT7 (0.696), PRSS3 (0.652) (Fig. 6C).

**Table 2:** Multivariate COX regression analysis results of 5-gene signature

Gene	coefficient	HR	95% CI	P
ANKRD22	0.19956	1.22	0.96–1.55	0.098033
ARNTL2	0.5387671	1.71	1.19–2.46	0.003633
DSG3	0.141913	1.15	1.01–1.31	0.029027
KRT7	0.17315	1.19	0.97–1.46	0.093604
PRSS3	0.2050587	1.23	1.04–1.44	0.012608

As noted above, these results suggested that 5-mRNA signature model was able to effectively predict the survival of pancreatic patients in the training dataset (TCGA-PAAD). The 5-mRNA signature model was better than the single gene prediction model.

### External validation of 5-mRNA signature model

**Fig. 7:**
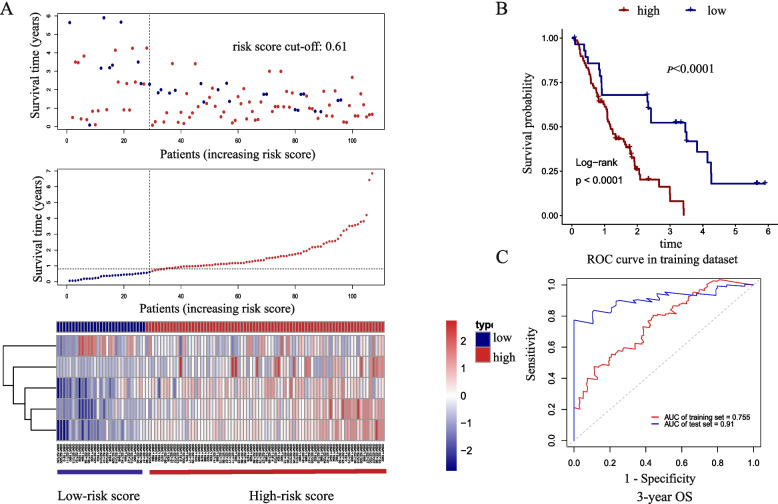
External validation of the 5-gene signature in GSE dataset (combined GSE62452 and GSE28735). **A** Patients in GSE dataset were divided into high-risk score and low-risk score groups according to the risk scores calculated with 5-gene signature. **B** Kaplan–Meier analysis estimates the OS of high-risk and low-risk groups in validation dataset. **C** ROC curves for 3-year OS by the 5-gene signature in training and validation dataset

Two GEO datasets, GSE62452 and GSE28735, were used for external validation. The sample size of a single dataset is limited. To improve the prediction accuracy, the two datasets were combined after removing batch effects. Risk scores were calculated with the same formula for each patient. The best cut-off value was calculated based on Kaplan–Meier analysis (supplementary Fig. 1B). Patients were divided into high-risk (*n* = 78) and low-risk (*n* = 29) groups according to the optimal cut-off value (Fig. 7A). Kaplan–Meier analysis with the 5-mRNA signature was used to compare the survival outcomes of patients in high-risk and low-risk groups. As shown in Fig. 7B, we confirmed that a higher risk score was associated with shorter survival time. We further analyzed the AUC value in the validation dataset, which was 0.91, significantly higher than AUC in the training dataset (0.755) (Fig. 7C).

In summary, the 5-mRNA signature's accuracy in predicting the prognosis of pancreatic cancer patients was validated, and the model performed better in the validation dataset.

## Discussion

In this study, we used TCGA and GEO datasets to construct 5-mRNA signature (ANKRD22, ARNTL2, DSG3, KRT7, PRSS3) that is associated with the prognosis of pancreatic cancer patients. The superiority of 5-mRNA signature was verified using the validation dataset. Our results suggested that patients with higher risk score that calculated on 5-mRNA signature, had shorter survival time.

Ankyrin repeat domain 22 (ANKRD22), a novel mitochondrial membrane protein. Several studies show that the expression of ANKRD22 is significantly elevated in various tissues and cells. Such as macrophages of patients with an acute rejection reaction after a renal transplant [8], peripheral blood mononuclear cells of pancreatic cancer patients [9], basal type I basal-like breast cancer tissues [10], non–small cell lung cancer (NSCLC) tissues [11]. In colorectal cancer cells, ANKRD22 plays a role in promoting glycolysis and reducing ATP levels [12]. Several studies suggest that the expression level of ANKRD22 is related to prognosis of pancreatic cancer [13], endometrial carcinoma [14], hepatocellular carcinoma [15].

Aryl hydrocarbon receptor nuclear translocator like 2 (ARNTL2), which encodes a basic helix–loop–helix transcription factor, is a member of PAS (PER ARNT, SIM) superfamily. ARNTL2 plays a role in biological processes like hypoxia and circadian [16]. Serval malignant tumors, including lung adenocarcinoma [17], colorectal cancer [18], breast cancer [19], are associated with dysfunction of ARNTL2.

Desmoglein 3 (DSG3) is an adhesion protein in desmosomes and is a member of the cadherin superfamily. Recent studies identify that DSG3 is a key role in several pathways, like cell adhesion and proliferation, morphogenesis, differentiation and migration [20, 21]. Recent evidences suggest that DSG3 might play an important role in the prognostic assessment of head and neck squamous cell carcinoma [22], skin cutaneous melanoma [23], and triple negative breast cancer [24].

Integrin subunit beta 6 (ITGB6), as one of Integrins family, has an increased expression level in some biological processes like wound healing, fibrosis, and malignant tumor formation [25]. ITGB6 regulates many basic pathways of the cell, such as ECM degradation, proliferation [26]. ITGB6 tends to be identified as an oncogene, which is upregulated in several solid tumors, and is associated with poorer prognosis and increased invasiveness [27].

Thyrotropin releasing hormone degrading enzyme (TRHDE), the only downregulated gene in 5-mRNA signature, the protein translated by it, has the function as extracellular inactivation of TRH (Thyrotropin releasing hormone) [28]. However, the role in cancer has not been elucidated. Only limited studies have demonstrated its role in tumor prognosis [29].

In clinical work, clinicians tend to use tumor TMN stage to evaluate the prognosis of pancreatic cancer patients. With the development of imaging technology, prognostic assessment combined with imaging data is also a feasible method. In any case, the current prognostic assessment method requires a high level of diagnosis capability for doctors and requires a certain amount of time for learning and training. At the beginning of the project, we wanted to find a simple, low-cost, universally adaptable way to perform prognostic assessment. Our prognostic assessment model includes 5 genes, making it easier and cheaper to test. By calculating the risk score, it is easier for clinicians to assess the prognosis of patients, to make clinical decisions and drug selection.

Since the prognosis evaluation of pancreatic cancer patients is important for the treatment of pancreatic cancer patients, many studies have focused on the role of prognosis-related genes in the prognosis evaluation of pancreatic cancer. Luo et al. identified 7-gene signature (ARNTL2, DSG3, PTPRR, ANLN, S100A14, ANKRD22, and TSPAN7) by using of TCGA, ICGC and GEO datasets. The assessment of 7-gene signature was carried out using ROC curves, which is same to our study [13]. Wu et al. conducted a 5-gene signature (AADAC, DEF8, HIST1H1C, MET, and CHFR) which was potential molecular targets for overall surviving of resectable pancreatic cancer patients [30]. Not only gene expression data, but DNA methylation data can also be used to evaluate the prognosis of pancreatic cancer patients, and it has achieved good results in the prognosis evaluation of pancreatic cancer patients [31]. On the other hand, noncoding RNA expression data can also be used to assess the prognosis of pancreatic cancer patients with the same accuracy [32]. Our study not only used lasso regression and multivariate Cox regression commonly used by other researchers, but also used a Gaussian mixed model to further screen variables. The results of our study were evaluated using the ROC curve and showed that 5-gene signature had good performance in both the training set and the validation set. The comparison of AUC values showed that our 5-gene signature was superior and comparable to previous studies and has not been reported by other researchers yet. This shows that our prognostic gene screening method is superior and provides a better model for pancreatic cancer prognosis evaluation.

However, our study also has limitations, the ultimate of which is that we did not use a larger external validation dataset for testing, all validation datasets are from public databases. In addition, we did not explore the biological functions of the five genes, which need to be verified by further in vivo and in vitro experiments, which is also the focus of our future research.

## Conclusion

Our study used a novel prognostic-related gene screening method and identified 5-gene signature as a prognostic assessment model for pancreatic cancer patients. This 5-gene signature performed well on both our chosen training dataset and validation dataset. These results provided a new way to predict the prognosis of pancreatic cancer patients.

## Supplementary Information

**Additional file 1.**

**Additional file 2.**

**Additional file 3.**

## Data Availability

The expression data of TCGA pancreatic cancer patients were downloaded from UCSC Xena (https://xenabrowser.net/datapages/). GTEx pancreatic cancer expression data was downloaded from UCSC Xena (https://xenabrowser.net/datapages/). GSE62452 and GSE28735 were downloaded from Gene Expression Omnibus database (https://www.ncbi.nlm.nih.gov/geo/).
