# Supplementary material for: Prognostic assessment capability of a five-gene signature in pancreatic cancer: a machine learning based-study
Source: BMC Gastroenterol. 2023 Mar 11;23:68. doi: 10.1186/s12876-023-02700-y (PMC10007739; doi:10.1186/s12876-023-02700-y)
Supplement: Supplementary file 1 — Additional file 1. [file 12876_2023_2700_MOESM1_ESM.pdf]

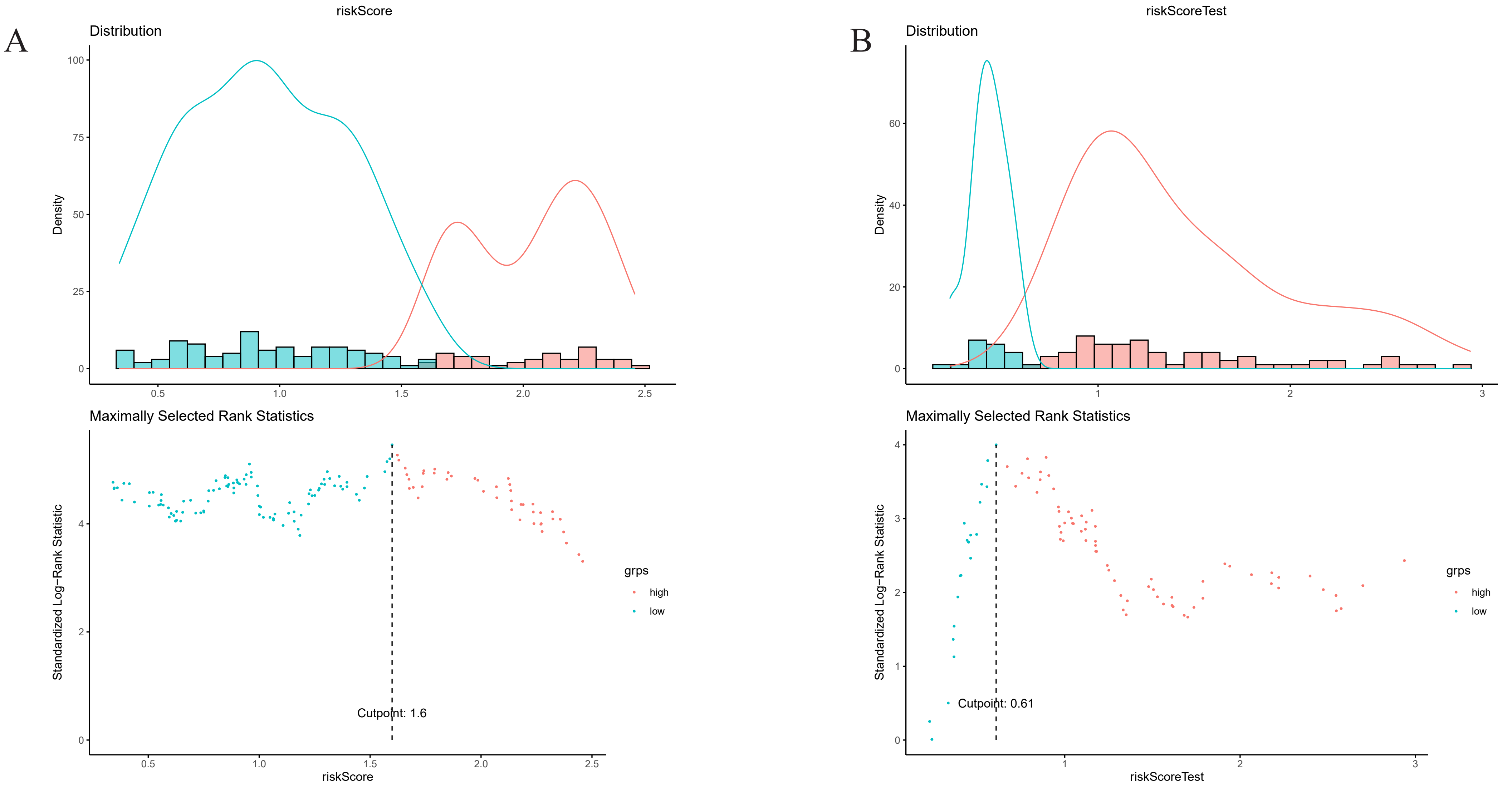

Supplementary figure 1: Pancreatic cancer patients were divided into high-risk and low-risk groups according to the cut-off values which were calculated with Kaplan-Meier method. (A) Cut-off value in training dataset (1.6). (B) Cut-off value in validation dataset (0.61).
