# Supplementary figures and images for: Prognostic assessment capability of a five-gene signature in pancreatic cancer: a machine learning based-study
Source: BMC Gastroenterol. 2023 Mar 11;23:68. doi: 10.1186/s12876-023-02700-y (PMC10007739; doi:10.1186/s12876-023-02700-y)

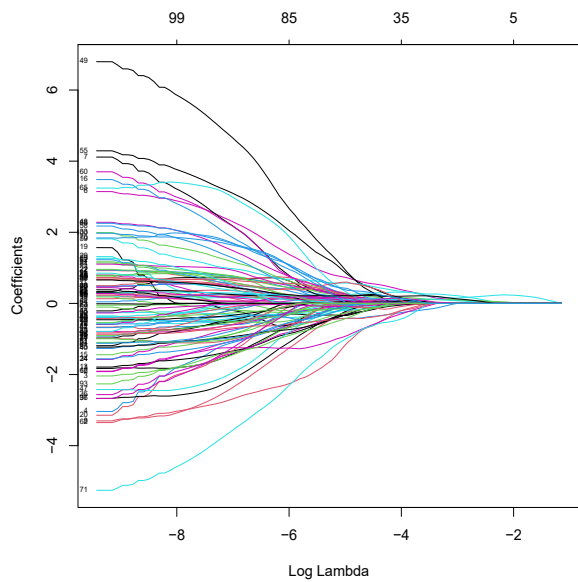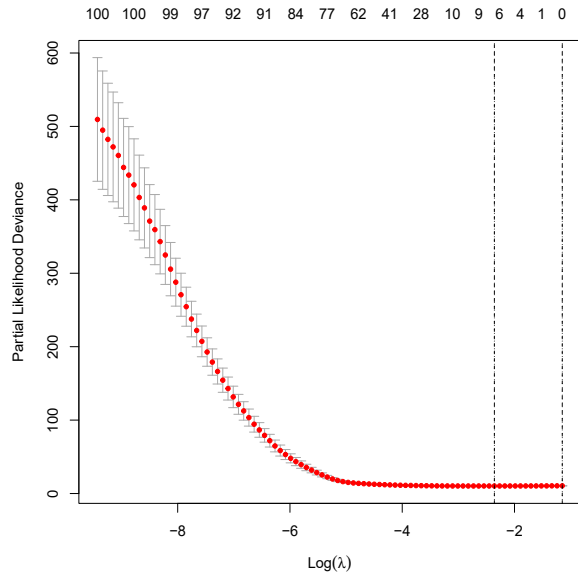

Supplementary figure 2: Lasso regression for variable screening.

Supplement: Supplementary file 2 — Additional file 2. [file 12876_2023_2700_MOESM2_ESM.pdf]
