# Supplementary material for: Prognostic assessment capability of a five-gene signature in pancreatic cancer: a machine learning based-study
Source: BMC Gastroenterol. 2023 Mar 11;23:68. doi: 10.1186/s12876-023-02700-y (PMC10007739; doi:10.1186/s12876-023-02700-y)
Supplement: Supplementary file 3 — Additional file 3. [file 12876_2023_2700_MOESM3_ESM.pdf]

Supplementary table 1: The table of Gaussian mixture model cluster analysis result.

model	auc	n.mrna	mclust
1	0.654729	1	1
2	0.687772	1	1
3	0.670508	1	1
4	0.663853	1	1
5	0.695819	1	1
6	0.691824	1	1
7	0.651644	1	1
8	0.584075	1	1
9	0.698601	2	1
10	0.70594	2	1
11	0.677292	2	1
12	0.710652	2	1
13	0.696865	2	1
14	0.671843	2	1
15	0.679384	2	1
16	0.696461	2	1
17	0.691598	2	1
18	0.718449	2	1
19	0.687965	2	1
20	0.719061	2	1
21	0.681336	2	1
22	0.68734	2	1
23	0.693239	2	2
24	0.705597	2	2
25	0.711084	2	2
26	0.682727	2	1
27	0.701125	2	2
28	0.687688	2	2
29	0.687375	2	2
30	0.66104	2	1
31	0.702332	2	2
32	0.712377	2	2
33	0.690884	2	2
34	0.734973	2	4
35	0.687017	2	2
36	0.646371	2	1
37	0.714483	3	2
38	0.694672	3	2
39	0.726839	3	2
40	0.698804	3	2
41	0.72743	3	2
42	0.696283	3	2
43	0.706116	3	2
44	0.714176	3	2
45	0.70745	3	2
46	0.726985	3	2
47	0.717334	3	2
48	0.706039	3	2
49	0.70207	3	2
50	0.686179	3	2
51	0.676932	3	2
52	0.717674	3	2
53	0.71724	3	2
54	0.706652	3	2
55	0.727551	3	2
56	0.693127	3	2
57	0.690628	3	2
58	0.694626	3	2
59	0.712241	3	2
60	0.702527	3	2
61	0.736817	3	4
62	0.697047	3	2
63	0.703265	3	2
64	0.693619	3	2
65	0.7289	3	2
66	0.681052	3	2
67	0.703302	3	2
68	0.743617	3	4
69	0.701689	3	2
70	0.741541	3	4
71	0.690167	3	2
72	0.715671	3	2
73	0.700659	3	2
74	0.708459	3	2
75	0.69812	3	2
76	0.68865	3	2
77	0.708334	3	2
78	0.714843	3	2
79	0.684533	3	2
80	0.737373	3	4
81	0.706032	3	2
82	0.719959	3	2
83	0.699843	3	2
84	0.713832	3	2
85	0.683832	3	2
86	0.730886	3	4
87	0.686249	3	2
88	0.67803	3	2
89	0.738657	3	4
90	0.692246	3	2
91	0.704324	3	2
92	0.731277	3	4
93	0.709845	4	2
94	0.723114	4	2
95	0.712402	4	2
96	0.736118	4	4
97	0.716326	4	2
98	0.716104	4	2
99	0.702283	4	2
100	0.738795	4	4
101	0.696267	4	2
102	0.715508	4	2
103	0.750291	4	4
104	0.720186	4	2
105	0.743154	4	4
106	0.69713	4	2
107	0.734544	4	4
108	0.715887	4	2
109	0.709213	4	2
110	0.706736	4	3
111	0.701736	4	3
112	0.71929	4	2
113	0.726033	4	4
114	0.70827	4	3
115	0.734373	4	4
116	0.716509	4	3
117	0.729064	4	4
118	0.713365	4	3
119	0.70891	4	3
120	0.702347	4	3
121	0.726077	4	4
122	0.696373	4	3
123	0.697997	4	3
124	0.738784	4	4
125	0.703672	4	3
126	0.717529	4	3
127	0.728916	4	4
128	0.702338	4	3
129	0.704821	4	3
130	0.736667	4	4
131	0.696143	4	3
132	0.712386	4	3
133	0.74625	4	4
134	0.696568	4	3
135	0.74617	4	4
136	0.705975	4	3
137	0.733566	4	4
138	0.705828	4	3
139	0.739899	4	4
140	0.697198	4	3
141	0.739767	4	4
142	0.688947	4	3
143	0.731693	4	4
144	0.744402	4	4
145	0.692246	4	3
146	0.74317	4	4
147	0.743511	4	4
148	0.709128	4	3
149	0.711323	4	3
150	0.685091	4	3
151	0.736305	4	4
152	0.70379	4	3
153	0.695806	4	3
154	0.740155	4	4
155	0.699725	4	3
156	0.713345	4	3
157	0.740282	4	4
158	0.736998	4	4
159	0.688465	4	3
160	0.702306	4	3
161	0.728236	4	4
162	0.738869	4	4
163	0.720822	5	3
164	0.709092	5	3
165	0.740915	5	4
166	0.713635	5	3
167	0.723362	5	3
168	0.754651	5	4
169	0.71776	5	3
170	0.743345	5	4
171	0.713167	5	3
172	0.74035	5	4
173	0.71326	5	3
174	0.749745	5	4
175	0.71591	5	3
176	0.740499	5	4
177	0.699944	5	3
178	0.736481	5	4
179	0.749588	5	4
180	0.706604	5	3
181	0.754086	5	4
182	0.748573	5	4
183	0.719242	5	3
184	0.71768	5	3
185	0.708526	5	3
186	0.73375	5	4
187	0.71369	5	3
188	0.713202	5	3
189	0.73823	5	4
190	0.718585	5	3
191	0.71957	5	3
192	0.741318	5	4
193	0.739135	5	4
194	0.703831	5	3
195	0.711958	5	3
196	0.729347	5	4
197	0.734185	5	4
198	0.711032	5	3
199	0.74306	5	4
200	0.693696	5	3
201	0.741916	5	4
202	0.704001	5	3
203	0.732025	5	4
204	0.747242	5	4
205	0.698377	5	3
206	0.741758	5	4
207	0.743966	5	4
208	0.742428	5	4
209	0.692232	5	3
210	0.738648	5	4
211	0.73948	5	4
212	0.74558	5	4
213	0.738352	5	4
214	0.699936	5	3
215	0.706619	5	3
216	0.736222	5	4
217	0.738858	5	4
218	0.737596	5	4
219	0.721274	6	3
220	0.751748	6	4
221	0.717134	6	3
222	0.741916	6	4
223	0.711378	6	3
224	0.741487	6	4
225	0.753494	6	4
226	0.717913	6	3
227	0.750175	6	4
228	0.743647	6	4
229	0.751538	6	4
230	0.706614	6	3
231	0.751931	6	4
232	0.742662	6	4
233	0.749908	6	4
234	0.737929	6	4
235	0.718429	6	3
236	0.719231	6	3
237	0.736642	6	4
238	0.737818	6	4
239	0.734713	6	4
240	0.742956	6	4
241	0.701867	6	3
242	0.740172	6	4
243	0.742046	6	4
244	0.745471	6	4
245	0.745548	6	4
246	0.738088	6	4
247	0.752571	7	4
248	0.717821	7	3
249	0.75064	7	4
250	0.743606	7	4
251	0.750017	7	4
252	0.750289	7	4
253	0.739684	7	4
254	0.744572	7	4
255	0.750519	8	4
